# pH-Dependent Interaction between C-Peptide and Phospholipid Bicelles

**DOI:** 10.1155/2012/185907

**Published:** 2012-07-16

**Authors:** Sofia Unnerståle, Lena Mäler

**Affiliations:** Department of Biochemistry and Biophysics, Center for Biomembrane Research, The Arrhenius Laboratories for Natural Sciences, Stockholm University, 106 91 Stockholm, Sweden

## Abstract

C-peptide is the connecting peptide between the A and B chains of insulin in proinsulin. In this paper, we investigate the interaction between C-peptide and phospholipid bicelles, by circular dichroism and nuclear magnetic resonance spectroscopy, and in particular the pH dependence of this interaction. The results demonstrate that C-peptide is largely unstructured independent of pH, but that a weak structural induction towards a short stretch of *β* -sheet is induced at low pH, corresponding to the isoelectric point of the peptide. Furthermore, it is demonstrated that C-peptide associates with neutral phospholipid bicelles as well as acidic phospholipid bicelles at this low pH. C-peptide does not undergo a large structural rearrangement as a consequence of lipid interaction, which indicates that the folding and binding are uncoupled. *In vivo*, local variations in environment, including pH, may cause C-peptide to associate with lipids, which may affect the aggregation state of the peptide.

## 1. Introduction

50 years ago, it was discovered that insulin is synthesized as proinsulin, which contains not only the two chains of insulin, A and B, but also a linker peptide, called C-peptide [1, 2]. C-peptide connects the two chains of insulin, which facilitates the disulfide bond formation between them and aids the folding process of insulin [3, 4]. Since the discovery, several biological effects of C-peptide have been demonstrated [5–7]. 

The primary structure of C-peptide varies significantly between different species, although certain common structural features can be observed. For example, the highly acidic and somewhat conserved N-terminus has properties that appear to be important for C-peptides chaperon-like effects on insulin disaggregation [8]. Further, the C-terminus is somewhat conserved and is likely to be involved in receptor interactions [9–11]. Human C-peptide, which is studied in this paper, consists of 31 aminoacid residues, EAEDLQVGQVELGGGPGAGSLQPLALEGSLQ. It contains many negatively charged amino acid residues and no basic residues resulting in a very low pI (3.5).

Human C-peptide has a random coil structure in buffer, while the N-terminal, third of the C-peptide (residues 1–11), has been demonstrated to be helical in 95% TFE [12]. In H_2_O/TFE 1 : 1, on the other hand, it has been shown that residues A2 through L5 adopt a type I *β*-turn, while residues E27 through Q31, the so-called pentapeptide, is the most ordered part of C-peptide adopting a type III′  *β*-turn [13]. Further, residues Q9-L12, residues G15-A18 and residues Q22-A25 were all shown to have structural preferences in the NMR-derived ensemble average [13]. It has recently been demonstrated that C-peptide also has the ability, under certain conditions, such as low pH, to form *β*-sheet structure, resembling amyloid structures [14, 15]. The peptide forms predominantly low-order oligomers [14], but very low concentrations of amyloid-like structures may also form [15]. The formation of amyloid structure can be enhanced in the presence of subcritical micelle concentration (CMC) amounts of SDS (at low pH), while SDS in amounts above the CMC, on the other hand, promote a more *α*-helical structure [15]. 

Even though C-peptide appears to only be marginally structured in aqueous solution and in solvents such as TFE, its ability to transiently adopt a variety of structures appears to be of importance for the peptides aggregation propensities. The ability of peptides and proteins to self-associate has been recognized in several diseases, including Alzheimer's disease, amyotrophic lateral sclerosis, and type II diabetes [16, 17]. In many cases, it has been demonstrated that the membrane may serve as a means for peptides to undergo structural rearrangements, which may be important for misfolding events. For the APP A*β* peptide, the composition of the membrane has been shown to be crucial for formation of amyloid structure [18–20]. Due to this feature of the peptide, and its previously demonstrated interaction with SDS, we have in this study examined the interaction between C-peptide and membrane mimetic media composed of isotropic phospholipid bicelles [21–26]. To compare, we have also examined the structure of C-peptide in different large unilamellar vesicle solvents. Further, since it has been demonstrated that pH is an important factor that governs the aggregation state of C-peptide [15], much like for the APP A*β* peptide [27] we have investigated the effect of pH on its structure and lipid interaction properties. In this way, the basic biophysical properties of C-peptide have been deduced, and this study shows that pH affects the ensemble average of the structure of C-peptide, which in turn affects the interaction with membrane-mimicking systems. 

## 2. Materials and Methods

### 2.1. Materials and Sample Preparation

C-peptide was purchased from PolyPeptide Laboratories, France and used without further purification. 1-palmitoyl-2-oleoyl-*sn*-glycero-3-phosphocholine (POPC) and 1-palmitoyl-2-oleoyl-*sn*-glycero-3-phospho-(1′-rac-glycerol)(POPG) were used to produce large unilamellar vesicles (LUVs). Deuterated lipids, 1,2-dihexanoyl-d_22_-sn-glycero-3-phosphocholine (d_22_-DHPC), 1,2-dimyristoyl-d_54_-sn-glycero-3-phosphocholine (d_54_-DMPC) and 1,2-dimyristoyl-d_54_-sn-glycero-3-phospho-(1′-rac-glycerol) (d_54_-DMPG) were used to produce bicelles. All phospholipids were obtained from Avanti Polar Lipids (Alabaster, AL, USA). Different buffers were used to study how the pH affects the C-peptide, three sodium phosphate buffers of pH 5.8, 6.9, and 7.2 and one citrate buffer of pH 3.2. 

Large unilamellar vesicles (LUVs) were produced for studying the interaction between C-peptide and bilayers by circular dichroism (CD). First, 20 mM stock solutions of neutral and 50% negatively charged vesicles were prepared by dissolving POPC and POPC/POPG 1 : 1, respectively, in chloroform. The samples were then dried under a flow of N_2_ gas to create lipid films. To ensure that no chloroform remained, the samples were stored under vacuum overnight. The dried lipid films were then soaked in buffer and vortexed for 10 minutes to obtain a more defined size distribution. The solutions were subsequently subjected to five freeze-thaw cycles to decrease lamellarity. Finally, to obtain uniform samples of LUVs, the samples were extruded around 20 times through a polycarbonate microfilter with 100 nm pore size. The CD samples were then prepared from these stock solutions and from stock solutions of 100 *μ*M C-peptide to a final concentration in the CD samples of 50 *μ*M C-peptide and 1 mM POPC or POPC/POPG 1 : 1 in 50 mM buffer (citrate buffer at pH 3.2 and sodium phosphate buffer at pH 5.8 or pH 6.9).

Small isotropic bicelles were used to further investigate membrane interaction of C-peptide by diffusion NMR and 2D total correlation spectroscopy (TOCSY). For samples used in pulse field gradient (PFG) diffusion measurements, the peptide was initially dissolved in buffer solution (D_2_O), and lipids were added to each solution. Buffer solutions of pH 3.2, 5.8, and 7.2, prepared from 50 mM citrate buffer (pH 3.2) or sodium phosphate buffer (pH 5.8 or pH 7.2) were dried and subsequently dissolved in D_2_O. 250 *μ*M C-peptide was dissolved in each buffer by sonication in a water bath for 1 min. PFG NMR experiments were acquired to measure the self-diffusion of C-peptide, *D*_free_. Subsequently d_54_-DMPC lipids and d_22_-DHPC dissolved in D_2_O were added in a ratio of 1 : 2 to each of the three different samples. This resulted in a final total lipid concentration of 150 mM and a q-ratio (long-chain phospholipids/short-chain phospholipids) of 0.5. After spectra were recorded for these samples, DMPG was added to the three samples, corresponding to 10% of the total long-chain lipids. In this way, bicelles with 10% negative charge were obtained. For 2D TOCSY NMR measurements, 200 *μ*M peptide was dissolved in DMPC/DHPC or in (DMPC/DMPG 9 : 1)/DHPC, respectively. Here, the total lipid concentration was 300 mM, and the q-ratio was 0.25. All bicelle mixtures were vortexed until a clear low-viscous solution was formed.

### 2.2. Circular Dichroism

The measurements were acquired on a Chirascan CD spectrometer with a 1 mm quartz cell for samples with 50 *μ*M peptide content and 1 mM POPC or POPC/POPG 1 : 1. The temperature was adjusted to 298 K with a TC 125 temperature control. Wavelengths ranging from 190 to 250 nm were measured with a 0.5 nm step resolution. Spectra were collected and averaged over ten measurements. Background spectra of buffers, POPC and POPC/POPG 1 : 1 without any peptide, were also recorded and were subtracted from the peptide spectra. 

### 2.3. NMR Spectroscopy

Translational diffusion experiments were performed on a Bruker Avance spectrometer, equipped with a triple resonance probe-head and operating at a ^1^H frequency of 600 MHz. A standard sample of 0.01% H_2_O in D_2_O, with 1 mg/mL GdCl_3_ to avoid radiation damping, was used for calibration of the gradient strength. The temperature was adjusted to 298 K using d_4_-methanol. Diffusion constants were measured using a modified Stejskal-Tanner spin-echo experiment [28–30] using a fixed diffusion time (300 ms) to minimize the influence of relaxation contributions, and a fixed gradient length (2.4 ms in buffer, 3 ms in bicelle solution, and 3.4 ms in acidic bicelle solution) and with a gradient strength varying linearly over 32 steps. The linearity of the gradient was calibrated as described previously [31]. The diffusion coefficient for HDO was measured and compared to the standard diffusion of HDO in D_2_O (1.9 10^−9^) [32]. This ratio was then multiplied to all measured diffusion constants to correct for viscosity differences induced by the sample.

2D TOCSY experiments [33] were recorded on Bruker Avance spectrometers operating at ^1^H frequencies of 500 MHz or 600 MHz at 298 K. TOCSY spectra were recorded with mixing times of 30 ms. Typically, 24–48 transients were recorded, and the number of increments in the indirect dimension was 256–512. The assignment of the spectra was achieved by the aid of the assignment by Munte et al. with the BMRB accession code 6623 [13], which was made at pH 7.0 and at 283 K. No chemical shift differences larger than ±0.03 ppm were seen between this assignment and our spectra obtained at pH 5.8 and at 298 K.

## 3. Results

### 3.1. C-Peptide Shows Overall Random Coil Features

**Figure 1:**
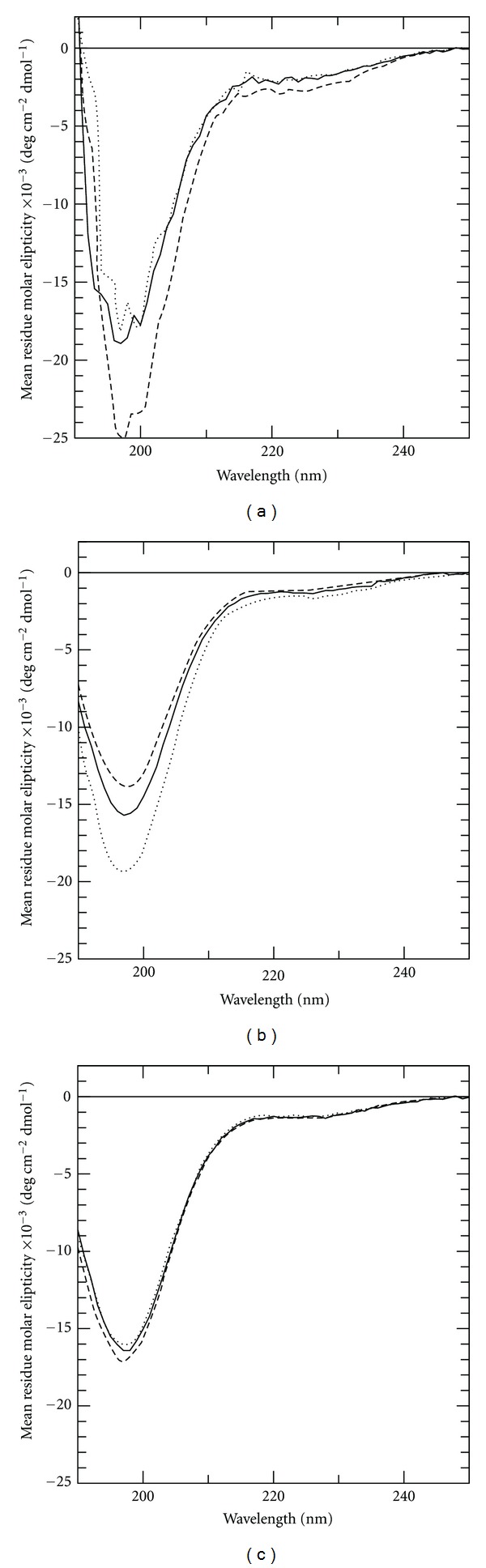
Secondary structure of C-peptide in LUVs at different pH as determined by CD. The figures depict CD spectra of 50 *μ*M C-peptide in buffer (dotted line), in 1 mM POPC (continuous line), and in 1 mM POPC/POPG 1 : 1 (dashed line). (a) Shows spectra for C-peptide in 50 mM citrate buffer, pH 3.2, (b) for C-peptide in 50 mM sodium phosphate buffer, pH 5.8, and (c) for C-peptide in 50 mM sodium phosphate buffer, pH 6.9.

Other studies have shown that C-peptide is predominately unstructured in aqueous solution and in the presence of lipid vesicles at pH 5 and 7 [12]. This is also seen in our CD spectra (Figures 1(b) and 1(c)), which show random coil features in buffered solutions, POPC and POPC/POPG 1 : 1, respectively. It has previously been shown that SDS induces oligomerization of C-peptide at a pH close to the pI of C-peptide (around 3.5) [15]. Thus, we wanted to study the structure of C-peptide at lower pH. Also at pH 3.2, C-peptide is predominantly in a random coil conformation and our results indicate that no major structural rearrangements are induced as a consequence of adding POPC or POPC/POPG 1 : 1, respectively (Figure 1(a)). The lack of structural induction indicates that there are no tight C-peptide-vesicle complexes formed, or that the peptide does not undergo large structural rearrangements in the presence of bilayers. However, local structural rearrangements or weak ensemble average preferences for the overall structure too small to be detected by far-UV CD may be significant for lipid interactions [34]. Such rearrangements may be detected in the H^N^-H^*α*^ region in TOCSY spectra, since the H^*α*^ chemical shifts are especially sensitive to local environment [35]. 

### 3.2. Chemical Shift Changes Reveal That Structural Rearrangements Are Induced by Lowering the pH

**Figure 2:**
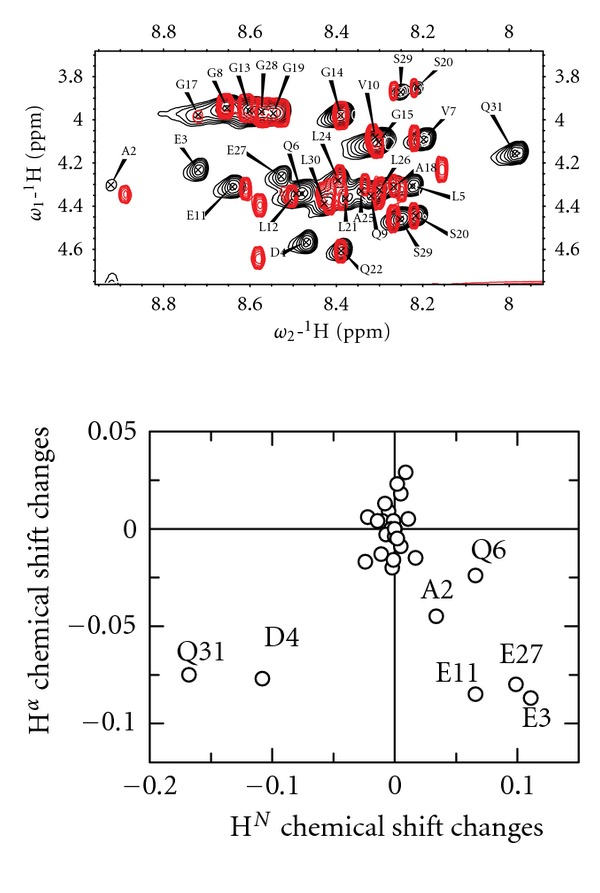
(a) 2D TOCSY spectra recorded at 600 MHz for 200 *μ*M C-peptide in 50 mM citrate buffer pH 3.2 (red) and in 50 mM sodium phosphate buffer pH 5.8 (black). (b) Differences in amide proton chemical shifts between pH 3.2 and pH 5.8 (i.e., amide proton shift pH 5.8 and amide proton shift pH 3.2) are plotted against differences in *α* proton chemical shifts (i.e., *α* proton shift pH 5.8 and *α* proton shift pH 3.2).

Even though the C-peptide is predominately unstructured at all pH studied in this paper as seen in CD spectra (Figures 1(a)–1(c)), lowering the pH was observed to induce a slightly different population average of structures as evidenced by small shift changes in 2D TOCSY spectra (Figure 2(a)). The peaks were generally observed to become less dispersed at pH 3.2 as compared to at pH 5.8. By comparing the chemical shifts under these two conditions, more detailed information can be gained (Figure 2(b)). The amino acid residues that show the greatest chemical shift differences between pH 3.2 and pH 5.8 are the terminal glutamine Q31, probably due to its greater solvent accessibility, and the negatively charged residues E3, D4, E11, and E27 (the N-terminal E1 is not visible in the spectra). These negatively charged amino acid residues are less likely to be charged at pH 3.2 (close to the pI of the C-peptide) due to protonation, which most likely is the main reason for the differences in chemical shifts. Other amino acid residues that are significantly affected by the change in pH are A2 and Q6. The H^*α*^ chemical shifts for both these residues are likely to be influenced by the change in environment of E1 and E3; nevertheless, significant chemical shift changes are observed for a large part of the N-terminus of C-peptide.

**Figure 3:**
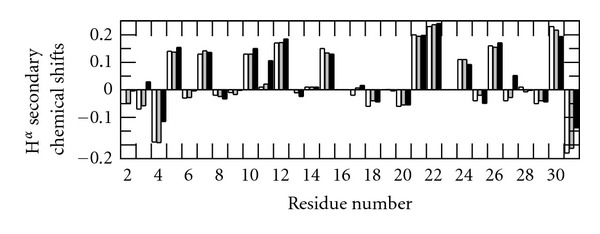
(a) H*α* secondary chemical shifts for each amino acid residue in C-peptide in pH 7.0 (white), 5.8 (grey), and 3.2 (black).

To investigate if these chemical shift differences are of importance for local structure induction, secondary chemical shifts were calculated for the H^*α*^ protons (Figure 3) [35]. When comparing the secondary chemical shifts at pH 3.2, 5.8, and 7, two stretches of amino acid residues are seen to move towards higher secondary chemical shift values with decreasing pH, residues A2 through Q6, and residues Q9 through L12, although the secondary chemical shift values are not consistently positive or negative in the first stretch of residues. These regions correspond well with two out of the five local structured regions previously found by Munte et al. in the C-peptide solution structure in H_2_O/TFE 1 : 1 [13]. Three amino acid residues not located in this region also have the same pattern, G17, Q22 and E27. Since most of these amino acid residues are located close to acidic residues, these shift changes can be induced by protonating the charged residues and are not necessarily due to a pH-induced structural rearrangement. In Figure 3, three residues in a row with a secondary chemical shift above 0.1 indicates *β*-sheet structure. Such a stretch is found at the lower pH (3.2), that is, V10-L12, indicating a tendency for *β*-sheet structure for this small part of the sequence. This corresponds well with previous studies, which identified residues Q9–L12 as being capable of forming *β*-turns in H_2_O/TFE 1 : 1 [13] and showed that lower pH is needed to induce *β*-structure, but then in the presence of SDS [15]. Here, we show that this small structural arrangement can be induced without any strong structural inducers like TFE or SDS, just by changing the pH. When correcting for nearest-neighbor effects according to Wishart et al. [36] (data not shown) G15, Q22, and L24 were the residues that were most affected, all moving to negative secondary chemical shifts with values larger than −0.1. 

In summary, lowering the pH induces small but significant chemical shift changes due to changed preferences in the population average. Some of these changes are most likely due to changes in the protonation state of the acidic residues, while a short N-terminal sequence as well as residues V10–L12 is seen to undergo small conformational changes towards more *β*-like structures.

### 3.3. Bicelles Induce Chemical Shift Changes at Low pH

**Figure 4:**
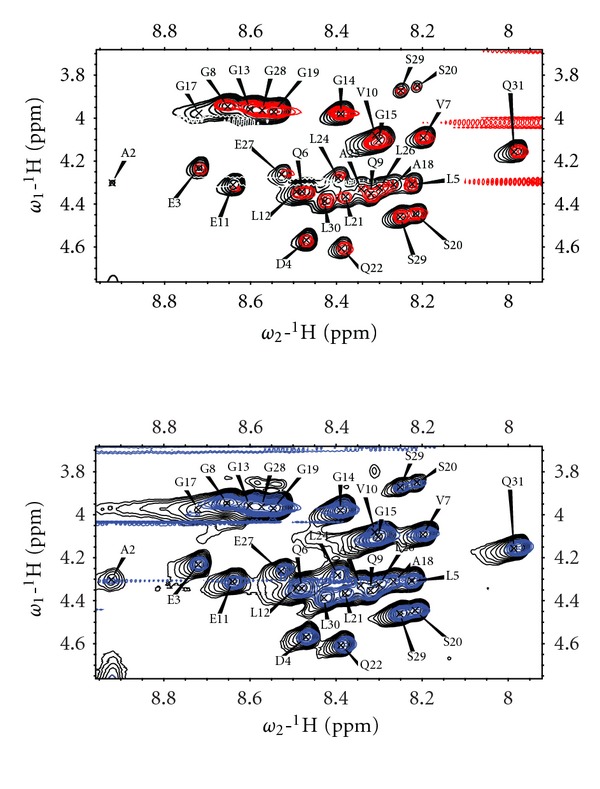
(a) 2D TOCSY spectra recorded at 600 MHz for 200 *μ*M C-peptide in bicelles (300 mM total lipid, *q* = 0.25) at pH 5.8. The spectra acquired in buffer are shown in black, while the spectra in bicelle solution are shown in (a) red for DMPC/DHPC bicelles and (b) blue for (DMPC/DMPG 9 : 1)/DHPC bicelles.

**Figure 5:**
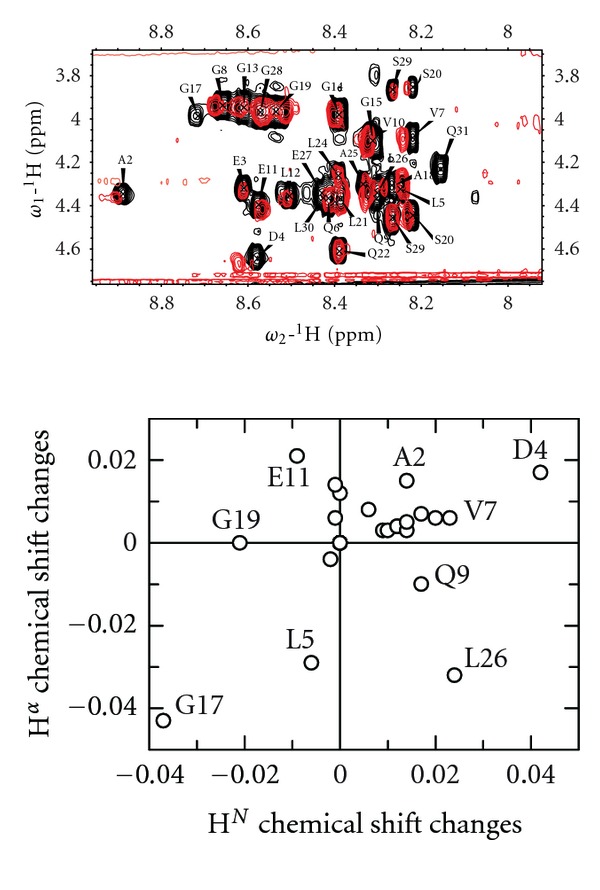
(a) 2D TOCSY spectra of 200 *μ*M C-peptide in buffer (black) and in DMPC/DHPC bicelles (300 mM total lipid, *q* = 0.25) (red) at pH 3.2. (b) Differences in amide proton chemical shifts between C-peptide in citrate buffer and C-peptide in DMPC/DHPC bicelles at pH 3.2 are plotted against differences in *α* proton chemical shifts (i.e., chemical shift in bicelles and chemical shift in buffer).

To investigate if the shift changes seen between pH 5.8 and pH 3.2 affect the membrane interaction, we examined changes in the TOCSY spectra when adding DMPC/DHPC bicelles [37, 38] with a *q*-ratio of 0.25 to 200 *μ*M peptide (giving a total lipid/peptide ratio of 1500 : 1) at pH 5.8 (Figure 4) and at pH 3.2 (Figure 5). No shift changes were seen upon adding bicelles at pH 5.8 (Figure 4(a)), and furthermore, no changes were seen when adding *q* = 0.25 (DMPC/DMPG 9 : 1)/DHPC bicelles either (Figure 4(b)). At this pH, C-peptide has a negative net charge; hence, the association with bicelles is not expected to increase by adding negative charges to the membrane. At pH 3.2, on the other hand, chemical shift changes are observed (Figure 5(a)). These chemical shift changes are not for the same residues that were identified as pH-sensitive. Rather, the shifts of A2, D4, L5, V7, Q9, E11, G17, G19, and L26 were affected by adding the bicelles (Figure 5(b)). Out of these residues A2, D4, V7, and E11 move to more *β*-sheet like shifts, while L5, Q9, G17, and L26 move to more *α*-helical shifts. The small stretch which was observed to adopt *β*-sheet-like shifts in buffer at pH 3.2 (V10–L12) became even more pronounced when adding bicelles. Hence, the interaction with bicelles stabilizes or favors this structure. Further, this shows that the previously reported structural preferences in C-peptide in TFE are seen also when using membrane mimetics, suggesting that this stretch may be of relevance *in vivo*, and may at least transiently interact with lipids.

In summary, we observed small but significant bicelle-induced chemical shift changes at pH 3.2 but not at pH 5.8, that stabilize the *β*-sheet structure of V10–L12.

### 3.4. PFG Diffusion Data Show That the Bicelle Association Is Greater at Lower pH

To investigate the extent of the C-peptide-lipid interactions, diffusion coefficients were measured in buffer and in the presence of different lipid bicelles. First, the self-diffusion coefficients were measured for C-peptide (250 *μ*M) in 50 mM phosphate buffer of pH 5.8 and 7.2 and in 50 mM citrate buffer of pH 3.2. These values are shown in Table 1. By using the relationship between the hydrodynamic radius (r_H_) and the molecular weight (M_r_) for nonstructured peptides (*r*_H_ = 0.27M_r_^0.50^) [39] and by using the theoretical molecular weight of the monomer (3020.3 Da), it became evident that the major population of C-peptide under all these conditions is that of a monomeric peptide (D = 1.66 · 10^−10^ m/s^2^). We, therefore, conclude that only changing the pH does not induce a detectable amount of oligomerization of C-peptide monomers. Second, the diffusion coefficients for C-peptide in phospholipid bicelles (150 mM total lipid, *q* = 0.5) with the bilayer part made of DMPC or DMPC/DMPG 9 : 1 were acquired. Measurements were carried out at similar pH values as used in the CD measurements. The diffusion coefficients for C-peptide decreased significantly when adding the phospholipid bicelles at all pH as seen in Table 1. The populations of free and bicelle-bound molecules can be estimated using a two-state model:

(1) xDDMPC+(1−x)Dfree=Dmixture,

where  *D*_free_ represents the diffusion of free peptide in solution (obtained from measurements in buffer), and *D*_DMPC_ is the diffusion coefficient for DMPC, that is, the diffusion rate of the bicelles. It has previously been demonstrated that all DMPC molecules are bicelle-bound, and hence it can be safely assumed that the diffusion coefficient for DMPC represents the diffusion of the bicelle [23–26]. Finally, *D*_mixture_ is the measured diffusion coefficient for C-peptide in the bicelle solution. This calculation suggests that around 65% of the peptide is bound to both DMPC and DMPC/DMPG bicelles at pH 3.2, when the C-peptide is neutral, while at pH 5.8 and 7.2 only 8–34% is bound, depending on the type of bicelle and pH. Hence, pH is important for the degree of association with the bicelles, and negatively charged C-peptide does not associate with the lipids to the same degree as C-peptide at low pH. As seen previously in the chemical shift analyses, charged lipids (DMPG in this case) do not appear to change the interaction.

## 4. Discussion

**Figure 6:**
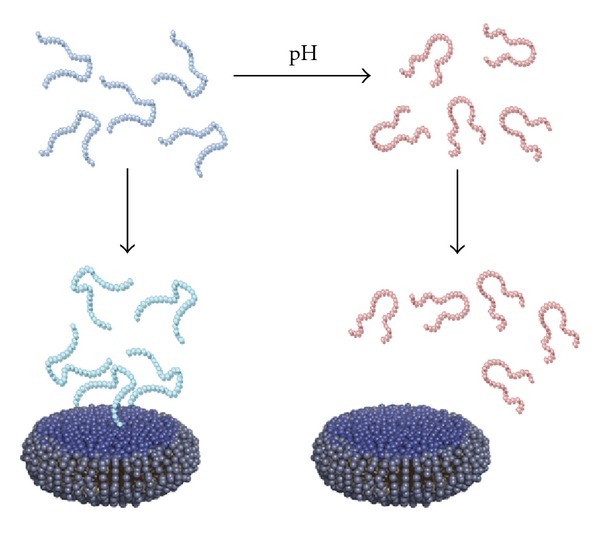
When lowering the pH to 3.2 (left panel), small structural rearrangements are induced that facilitate interaction with phospholipid bicelles. The interaction with bicelles further influences the structure. At pH 5.8, on the other hand, (right panel), no structural arrangements are seen upon addition of bicelles and the interaction is much weaker.

In this study, we wished to elucidate the effect of pH on C-peptide structure and lipid interactions. Previous studies have examined the effects of lipids on C-peptide structure at pH 5 or higher, and neither CD spectroscopy or size exclusion chromatography has revealed any lipid interaction [12]. Hence, it was then concluded that stable conformation-dependent interactions of C-peptide with lipid membranes are unlikely to occur. Biological effects of C-peptide, protecting against diabetic complications, are mediated by interaction with insulin or interaction with membrane via specific and/or nonspecific membrane interaction. Most studies support specific interactions with a, yet to be found, GPCR [40, 41]. However, the D-enantiomer of C-peptide has the same biological activity as the L-enantiomer [42], which suggests that other receptor-independent interactions are important for function. Formation of cation-selective channels in lipid bilayers [43] also suggests a more nonspecific interaction. Hence, we find it valuable to investigate nonspecific interactions between C-peptide and the membrane as a part of C-peptides protective function. We have previously demonstrated that, at low pH, C-peptide has the ability to form *β*-sheet-like aggregates at low detergent concentrations and *α*-helical structure in SDS micelles [15], indicating that pH is important for structural induction. Thus, the structure and lipid interaction of C-peptide was in the present study also examined at a lower pH close to the pI of the C-peptide. From the results, we see that C-peptide favors a lipid interaction at low pH, when the peptide is neutral, (around 65% of the peptide is associated with bicelles at pH 3.2), suggesting that the relationship between electrostatic and hydrophobic interactions is important for this process (Figure 6). By decreasing the pH, small structural rearrangements in predominately the N-terminal and in the amino acid stretch between V10 and L12 are induced, that facilitate lipid interaction. Upon addition of bicelles, these structural preferences are stabilized. The structural rearrangements of C-peptide, as judged from both CD and NMR spectroscopy are not large, and thus, C-peptide represents a group of membrane interacting peptides that do not appear to undergo large structural changes upon membrane binding. This behavior has previously been observed for, for example, the interaction between the opioid receptor peptide ligands (dynorphins) and bicelles, which did not cause any structural induction in the peptide ligands [34]. It appears that lack of structure induction is not a conclusive way of demonstrating lack of peptide-membrane interaction. Sometimes local and transient structural preferences in an ensemble of peptides dictate function [44]. This is similar to the recent findings that even protein-protein interactions may not always lead to well-formed secondary or tertiary structures, but indicates a novel mode of action of these intrinsically disordered proteins [45, 46].

Lipid-peptide interaction can further promote aggregation. It is well known that insulin forms oligomeric states and amyloid fibrils as a function of pH and ionic strength [19, 34–37]. However, C-peptide has also been demonstrated to form oligomers under conditions similar to* in vivo* situations, including sub-*μ*M concentrations [12, 13]. Although the interaction between C-peptide and lipids appears to be weak in the present study, the membrane can affect local concentrations of C-peptide and change the local pH, which can shift the equilibrium between C-peptide monomer and membrane bound species. For instance, the interfacial pH of anionic membranes can be much lower than the bulk pH. This may in turn have an effect on the aggregation of C-peptide. Local pH effect has previously been demonstrated to be of importance for, for example, membrane insertion of the pH (low) insertion peptide (pHLIP), where low pH promotes interaction with the membrane[47, 48]. 

Although earlier studies have demonstrated that interactions with lipid vesicle bilayers do not result in any membrane-induced structure conversion in C-peptide [12], local variations in environment, including pH, may cause C-peptide to associate with lipids, as demonstrated here, which may affect the aggregation state of the peptide, and the equilibrium between a dominating population of monomeric peptides and a very small population of oligomers may be shifted [14]. Hence, transient membrane interactions may be of importance *in vivo* for inducing aggregation without any apparent structure intermediate. 

## Figures and Tables

**Table 1:** Diffusion coefficients for the C-peptide.

pH	Diffusion coefficient (10^−10^ m^2^/s)^a^						
	Buffer	DMPC/DHPC			(DMPC/DMPG 4: 1)/DHPC		
	*D*_free_	*D*_mixture_	*D*_DMPC_	*x* (%)^b^	*D*_mixture_	*D*_DMPC_	*x* (%)^b^
3.2	1.5 ± 0.1	0.8 ± 0.1	0.48 ± 0.05	69	0.77 ± 0.1	0.36 ± 0.05	64
5.8	1.6 ± 0.1	1.4 ± 0.1	0.43 ± 0.05	17	1.1 ± 0.1	0.42 ± 0.05	34
7.2	1.5 ± 0.1	1.1 ± 0.1	0.34 ± 0.05	34	1.4 ± 0.1	0.25 ± 0.05	8

^a^The diffusion coefficients are normalized according to the diffusion of HDO to account for viscosity differences. ^b^Estimation of the percentage of peptide bound to the phospholipid bicelle as calculated by (1).

## Abbreviations

TFE: 2,2,2-Trifluoroethanol
SDS: Sodium dodecyl sulfate
CMC: Critical micelle concentration
APP: Amyloid precursor protein
POPC: 1-palmitoyl-2-oleoyl-sn-glycero-3-phospho-choline
POPG: 1-palmitoyl-2-oleoyl-sn-glycero-3-phospho-(1-rac-glycerol)
LUVs: Large unilamellar vesicles
DHPC: 1,2-dihexanoyl-d22-sn-glycero-3-phosphocholine
DMPC: 1,2-dimyristoyl-d54-sn-glycero-3-phosphocholine
DMPG: 1,2-dimyristoyl-d54-sn-glycero-3-phospho-(1-rac-glycerol)
CD: Circular dichroism
NMR: Nuclear magnetic resonance
TOCSY: Total correlation spectroscopy
PFG: Pulse field gradient
D: Diffusion coefficient
GdCl: Gadolinium(III) chloride
BMRB: Biological Magnetic Resonance Bank
M_r_: Molecular weight
r_H_: Hydrodynamic radii
GPCR: G protein-coupled receptor.
